# Risk factors of epilepsy recurrence in patients with post-stroke epilepsy

**DOI:** 10.12669/pjms.41.6.11519

**Published:** 2025-06

**Authors:** Xian Wang, Hongying Zhao, Liang Gao, Lan Zhang, Zhennan Li

**Affiliations:** 1 Xian Wang Department of General Internal Medicine, Baoding Third Central Hospital, 6-2-802 Shanshuihuating, Baoding, Hebei Province 071000, P.R. China; 2 Hongying Zhao Department of Neurology, Anxin County Hospital, Baoding, Hebei Province 071600, P.R. China; 3 Liang Gao Department of General Internal Medicine, Baoding Third Central Hospital, 6-2-802 Shanshuihuating, Baoding, Hebei Province 071000, P.R. China; 4 Lan Zhang Department of General Internal Medicine, Baoding Third Central Hospital, 6-2-802 Shanshuihuating, Baoding, Hebei Province 071000, P.R. China; 5 Zhennan Li Department of General Internal Medicine, Baoding Third Central Hospital, 6-2-802 Shanshuihuating, Baoding, Hebei Province 071000, P.R. China

**Keywords:** Anti-epileptic drugs, Epilepsy recurrence, Post-stroke epilepsy, Risk factors

## Abstract

**Objective:**

To explore the risk factors for epilepsy recurrence in patients with post-stroke epilepsy (PSE), and to determine the optimal timing of anti-epileptic drug administration.

**Methods:**

This retrospective study collected clinical data from 90 patients with post-stroke epilepsy (PSE) admitted to Baoding Third Central Hospital from February 2021 to December 2023. Patients were grouped based on epilepsy recurrence. Baseline data, including lesion characteristics, blood homocysteine levels, the National Institute of Health Stroke Scale (NIHSS) scores, and anti-epileptic drug therapy treatment effects, were compared between the recurrent and non-recurrent groups. Logistic analysis was done to determine the risk factors for epilepsy recurrence in patients with PSE.

**Results:**

Logistic analysis confirmed that stroke lesion located in the cortex, lesion size ≥ 3 cm, post-stroke late seizure, intracerebral hemorrhage (ICH), blood leakage into the surrounding infarcted tissue, hyperhomocysteinemia (HHcy), and NIHSS scores of ≥ 15 are risk factors for epilepsy recurrence in patients with PSE (P < 0.05). In the recurrent group, a statistically significant difference in treatment effect was found among patients who received anti-epileptic drugs at different time points (P < 0.05). The total efficacy was higher in the early-onset treated (90.91%), early-onset untreated (85.71%), and late-onset treated (80.00%) groups than in the late-onset untreated group (40.00%). In addition, the total effectiveness differed significantly between the early-onset treated and late-onset untreated groups (P < 0.05).

**Conclusion:**

Lesion location and size, timing of epileptic seizures, ICH, hemorrhagic transformation, HHcy, and NIHSS scores were identified as risk factors for epilepsy recurrence in patients with PSE. Although early-onset PSE does not require early anti-epileptic treatment, timely treatment should be administered in late-onset cases.

## INTRODUCTION

Post-stroke epilepsy (PSE) refers to a common complication of stroke when the epileptiform discharge arises from the area affected by the stroke.^1^ Approximately 10% of stroke patients develop epilepsy.^2^ High rates of disability and mortality associated with stroke present a serious public health problem and may significantly impact the quality of life and physical and mental health of survivors.^3^ The risk of epilepsy recurrence after a stroke is high, and it can not only worsen stroke symptoms but also potentially lead to vascular cognitive impairment and negatively impact the patients’ prognosis.^4^ Several studies have analyzed the risk factors for PSE, but there is considerable variation among the findings.^5^ Therefore, identifying the risk factors for the recurrence of seizures in PSE patients is crucial.

However, the treatment options for PSE are highly limited, and the optimal timing and type of anti-epileptic drugs therapy for the patients with PSE remain a contentious issue. It is also unclear which treatment methods are most effective in preventing symptom recurrence.^6^,^7^ The number of randomized controlled trials evaluating PSE and epileptic interventions is remarkably scarce. The few existing studies, due to their low quality of evidence, are unable to provide effective recommendations for the prevention and treatment of PSE.^8^. This retrospective study aimed to evaluate the risk factors for epilepsy recurrence and the optimal timing of anti-epileptic drugs use in patients with PSE.

## METHODS

We retrospectively identified 90 patients with PSE episodes at Baoding Third Central Hospital from February 2021 to December 2023, with each patient followed up for three months. Based on whether epilepsy recurred, patients were divided into a recurrence group and a non-recurrence group, with 43 in the recurrent and 47 patients in the non-recurrent group. Seizure recurrence after the first PSE episode was defined as epileptic recurrence (recurrence group), while no recurrence after the first PSE episode was defined as epileptic non-recurrence (non-recurrence group). Additionally, patients were categorized into early-onset and late-onset types based on the timing of seizure onset. Seizures occurring within seven days of stroke were defined as early-onset, while those occurring after seven days were defined as late-onset. Patients in the recurrent group were further divided into the early-onset treated, early-onset untreated, late-onset treated, and late-onset untreated subgroups based on the timing of anti-epileptic drugs therapy. All procedures performed in study involving human participants were in accordance with the ethical standards of the institutional and/or national research committee(s) and with the Helsinki Declaration (as revised in 2013).

### Ethical Approval:

This study has been approved by the Ethics Committee of Baoding Third Central Hospital (IRB No. 202301; Date: April 12, 2023). The informed consent was waived by the ethics committee for the observational and retrospective nature. All data were stored securely, and confidentiality was maintained throughout the study.

### Inclusion criteria:

- Met the diagnostic criteria for stroke,^9^ as confirmed using computed tomography and magnetic resonance imaging.
- Met the diagnostic criteria for epilepsy.^10^
- The first seizure occurred after the stroke.
- A clear correlation between epilepsy and stroke in terms of location and time.
- Complete clinical data.

### Exclusion criteria:

- Epilepsy caused by other factors.
- A history of epileptic seizures or suspected epilepsy before stroke.
- A family history of epilepsy.
- Metabolic disorders such as mitochondrial encephalopathy, hyponatremia, and abnormal blood glucose levels.
- Patient received quinolone antibiotics or other potentially epileptogenic drugs before inclusion in the study.
- Underlying conditions such as poisoning, shock, history of hand surgery, head trauma, intracranial infection, and intracranial tumors.
- A history of alcohol/addictive substance abuse or withdrawal reactions.

### Indicators collected:

- Baseline characteristics such as sex, age, smoking habits, drinking habits, stroke lesion location (cortical or subcortical), lesion size (divided into < 3 cm and ≥ 3 cm), type of epilepsy (early/late onset), type of stroke, and presence of hemorrhagic transformation after the stroke.
- Data regarding any history of coronary heart disease, diabetes, hypertension, hyperlipidemia, hyperhomocysteinemia (HHcy) and the neurological functional outcomes (assessed by the National Institutes of Health Stroke Scale [NIHSS]).^11^

### Evaluation of effectiveness:

Patients who did not experience any seizures during the three months follow-up period were considered to be seizure-free. A reduction in the frequency of seizures by ≥ 50% compared to the baseline level was considered to indicate effectiveness. Patients without seizures and those in whom the treatment was considered effective were included in the evaluation of the total effectiveness rate.

### Statistical analysis:

All analyses were conducted using SPSS software (version 25.0; IBM Corp, Armonk, NY, USA). The measurement data were reported as the mean ± standard deviation, and an independent samples t-test was used for inter-group comparison. Categorical variables were reported as frequencies and percentages, and differences between the three groups were assessed using chi-square tests. Logistic analysis was performed to determine the risk factors for epilepsy recurrence in patients with PSE; P values of < 0.05 were considered to indicate a statistically significant difference.

## RESULTS

No significant differences were observed between the two groups in terms of sex, age, smoking, alcohol intake, history of coronary heart disease, diabetes, hypertension, and hyperlipidemia (P > 0.05). However, the two groups significantly differed in lesion location and size, seizure type, intracerebral hemorrhage (ICH), hemorrhagic transformation, HHcy, and NIHSS scores (P < 0.05) (Table-I). Epilepsy recurrence was considered as the dependent variable, and the lesion location, lesion extent, type of seizure, ICH, oozing of blood in the brain, HHcy, and NIHSS score were considered as independent variables, and included into the logistic model. The assigned values are shown in Table-II. The results of the logistic model showed that the cortical location of the lesion, lesion size ≥ 3 cm, late-onset seizure type, ICH, leakage of blood in the surrounding brain tissue, HHcy, and NIHSS scores of ≥ 15 were risk factors for epilepsy recurrence in patients with PSE (P < 0.05) (Table-III).

**Table-I:** Comparison of baseline data between the two groups.

Baseline data		Recurrent group (n=43)	Non-recurrent group (n=47)	χ^2^/t	P
Gender	Male	24 (55.81)	28 (59.57)	0.130	0.718
	Female	19 (44.19)	19 (40.43)	
Age (years)		56.68±9.39	58.02±7.61	0.750	0.455
Smoking	Yes	18 (41.86)	23 (48.94)	0.453	0.501
	No	25 (58.14)	24 (51.06)		
Alcohol intake	Yes	20 (46.51)	25 (53.19)	0.401	0.527
	No	23 (53.49)	22 (46.81)		
Coronary heart disease	Yes	5 (11.63)	4 (8.51)	0.243	0.622
	No	38 (88.37)	43 (91.49)		
Diabetes	Yes	13 (30.23)	16 (34.04)	0.149	0.699
	No	30 (69.77)	31 (65.96)		
Hypertension	Yes	19 (44.19)	18 (38.30)	0.322	0.571
	No	24 (55.81)	29 (61.70)		
Hyperlipidemia	Yes	9 (20.93)	5 (10.64)	1.811	0.178
	No	34 (79.07)	42 (89.36)		
Location of lesion	Cortex	30 (69.77)	15 (31.91)	12.870	<0.001
	Subcortical	13 (30.23)	32 (68.09)		
Lesion size	<3 cm	18 (41.86)	35 (74.47)	9.862	0.002
	≥3 cm	35 (58.14)	12 (25.53)		
Seizure type	Early onset	13 (30.23)	32 (68.09)	12.870	<0.001
	Late-onset	32 (69.77)	15 (31.91)		
ICH	Yes	20 (46.51)	37 (78.70)	10.033	0.002
	No	23 (53.49)	10 (21.30)		
Hemorrhagic transformation	Yes	11 (25.58)	3 (6.38)	6.301	0.012
	No	32 (74.42)	44 (93.62)		
HHcy	Yes	12 (27.91)	2 (4.26)	9.563	0.002
	No	31 (72.09)	45 (95.74)		
NIHSS score	≥15	22 (51.20)	9 (19.10)	10.192	0.001
	<15	21 (48.80)	38 (80.90)		

ICH: intracerebral hemorrhage; HHcy: hyperhomocysteinemia; NIHSS: National Institutes of Health Stroke Scale.

**Table-II:** Assignment table.

Variable	Name	Assigned method
X1	Location of lesion	cortex=1, subcortical=2
X2	Lesion size	≥3 cm=1, <3 cm=2
X3	Seizure type	Late hairstyle=1, early hairstyle=2
X4	ICH	Yes=1, No=2
X5	Oozing of blood in the brain	Yes=1, No=2
X6	HHcy	Yes=1, No=2
X7	NIHSS score	≥15 points=1, <15 points=2

ICH: intracerebral hemorrhage; HHcy: hyperhomocysteinemia; NIHSS: National Institutes of Health Stroke Scale.

**Table-III:** Analysis of risk factors for epilepsy recurrence in PSE.

Variable	B	S.E.	Wald χ^2^	OR	P	95% CI
Lesion located in the cortex	3.939	1.324	2.97	51.368	0.003	1.344~6.534
Lesion size ≥ 3 cm	4.045	1.586	2.55	57.100	0.011	0.935~7.154
Late-onset epileptic seizure	3.159	1.457	2.17	23.539	0.030	0.303~6.015
ICH	2.524	1.163	2.17	12.483	0.030	0.245~4.804
Hemorrhagic transformation	2.618	1.152	2.27	13.704	0.023	0.360~4.875
HHcy	2.675	1.283	2.08	14.516	0.037	0.160~5.191
NIHSS score ≥ 15	4.024	1.901	2.12	55.939	0.034	0.299~7.750

***Notes:*** B indicates partial regression. S.E.: standard error; Wald χ2: (B/S.E.)2; OR: odds ratio; 95% CI: confidence interval of OR; ICH: intracerebral hemorrhage; HHcy: hyperhomocysteinemia; NIHSS: National Institutes of Health Stroke Scale.

A statistically significant difference was observed in the comparison between the spontaneous improvement of the recurrent group and the treatment effect of different anti-epileptic drug administration timings (P < 0.05). Among early-onset patients, the spontaneous improvement rate was 85.71% in the early-onset untreated group, while the effective treatment rate was 90.91% in the early-onset treated group, with a higher rate of complete seizure control in the treated group. Among late-onset patients, the spontaneous improvement rate was poorer in the late-onset untreated group (40.00%), whereas the effective treatment rate was higher in the late-onset treated group (80.00%). Whether for early or late onset seizures, the improvement and seizure-free were superior in the treated groups compared to the untreated groups (Table-IV).

**Table-IV:** Comparison of treatment effects among patients in the recurrent group who received anti-epileptic drugs at different time points.

Group	n	No seizures	Effective	Invalid	Total effective rate
Early-onset treated	11	8 (72.73)	2 (18.18)	1 (9.09)	10 (90.91)
Early-onset untreated	7	4 (57.14)	2 (28.57)	1 (14.29)	6 (85.71)
Late-onset treated	15	7 (46.67)	5 (33.33)	3 (20.00)	12 (80.00)
Late-onset untreated	10	1 (10.00)	3 (30.00)	6 (60.00)	4 (40.00)
*χ^2^*					8.509
*P*					0.037

## DISCUSSION

This retrospective study analyzed the risk factors for epilepsy recurrence in PSE patients, including stroke type, stroke lesion location, stroke lesion size, stroke severity (high NIHSS score), post-stroke seizure type, post-stroke hemorrhage, and HHcy.

Based on the mechanistic analysis of the identified risk factors, it is plausible that stroke-induced damage to the cortical layer may lead to an abnormal increase in excitability, as this layer has a dense network of cortical nerve cells (mainly composed of astrocytes, pyramidal cells, and axons) which have a relatively low threshold for epileptic seizures. This can increase the risk of epileptic seizures and promote the onset and progression of epilepsy.^2^–^5^ Therefore, patients with cortical lesions may be more prone to secondary seizures. A study of Ferreira-Atuesta et al.^12^ explored the risk factors for PSE and found a higher incidence of epilepsy in patients with stroke who had cortical involvement, which is consistent with our results. Notably, some studies have suggested that the subcortical layer has a higher threshold for abnormal discharge and is associated with a lower risk of epilepsy, which may be attributed to the fact that the subcortical tissue is mainly composed of white matter fibers and dendrites.^12^,^13^

Mechanistic analysis also suggests that larger stroke lesions are more likely to affect a larger number of blood vessels, leading in more severe cerebral hypoxia and ischemia.^12^–^14^ This may explain a higher risk of abnormal discharge observed in our cohort. Our findings are consistent with the observations of Lucci FR et al.^14^ Additionally, our study showed that the timing of epileptic seizures was an influencing factor of epilepsy recurrence in PSE patients. Early-onset epilepsy is often closely related to excessive secretion of neurotransmitters, metabolic disorders, and early ischemia after stroke.^15^ The metabolic disorders caused by stroke may therefore improve significantly after the acute phase. Notably, patients with early-onset epilepsy have a lower epilepsy recurrence rate.^15^,^16^ Özaydın Göksu E et al.^17^ suggested that late-onset seizures are often closely related to scar tissue formation, mechanical stimulation of lesions, and glial cell proliferation. The changes in these physiological and pathological factors do not disappear or weaken with an increase in stroke duration, thus explaining why patients with late-onset epilepsy are more prone to recurrence.^15^–^17^ Timely and effective treatments should therefore be provided in such cases.

The type of stroke was also closely associated with the recurrence of PSE in our study. Derex et al.^18^ has reported that in patients with ICH, the deposition and transformation of hemosiderin can lead to the production of large quantities of amino acids in local nerve cells, causing diffusion or focal cerebral vasospasm, and leading to electrolyte imbalance and depolarization of brain cells. These changes can considerably increase the likelihood of an abnormal discharge. Patients with cerebral hemorrhage therefore have a higher incidence of epilepsy compared to those with cerebral infarction, and the former has a long-term impact on epilepsy. Patients with hemorrhage are also at a higher risk of secondary epilepsy recurrence.^18^ Blood leak into the surrounding infarcted tissue is usually seen in patients with cardiogenic or extensive stroke. This type of stroke is more acute and can easily lead to interruption of cerebral blood flow, thereby causing severe hypoxia and ischemia of brain tissue. These changes ultimately lead to abnormal depolarization of neurons and trigger epilepsy.^19^,^20^ A study of Zelano et al.^20^ reported that blood-related metabolites formed after cerebral hemorrhage can exert certain stimulatory effects on local brain tissue, potentially increasing the to a certain extent the risk of epilepsy and secondary recurrence.

In this study, we identified HHcy as an important influencing factor for secondary seizures after stroke. The mechanism of this effect may be attributed to the varying degrees of damage that is caused to deoxyribonucleic acid and vascular endothelium by abnormally high levels of homocysteine. Such damage affects the microcirculation and brain function.^21^,^22^ Similarly to our results, a study by Tao et al.^22^ also reported that patients with epilepsy have abnormally high levels of homocysteine, with a significant correlation between the two variables.

Our results identified the significant association of NIHSS scores, which reflect the severity of clinical stroke symptoms, with the risk of seizure recurrence in PSE patients. Our observations are similar to the results of the population-based study by Zöllner et al.,^23^ which reported a proportional increase in the risk of acute symptomatic seizures with increasing NIHSS score at admission. Cortical involvement and larger lesion sizes were identified as critical factors associated with severe clinical symptoms. This finding is consistent with the results reported by Hsieh et al.^24^

Our findings suggest that early anti-epileptic treatment is unnecessary in cases of early-onset PSE. At the same time, patients with late-onset seizures would benefit from timely treatment. Similarly, Sun et al.^25^ found that risk factors for early-onset epilepsy following a stroke can usually be improved, and therefore, long-term anti-epileptic interventions are not necessary. However, late-onset epilepsy is closely associated with encephalomalacia and necrosis. It is caused by stroke sac traction, neuronal degeneration, and neuron-stimulated small vessel proliferation that may lead to abnormal discharges, which are often challenging to eliminate.^25^,^26^ Long-term standardized anti-epileptic treatment should, therefore, be administered in cases of late-onset epilepsy. In summary, this report identified risk factors for epilepsy recurrence in post-stroke epilepsy patients, which can be used clinically to identify high-risk populations for recurrence. Meanwhile, this study also confirmed that early-onset epilepsy after stroke does not require early antiepileptic treatment, while late-onset patients should receive timely antiepileptic treatment.

### Limitations:

Firstly, it is a retrospective study, which introduced a risk of a potential selection bias and limited our ability to establish causal relationships. Secondly, due to the retrospective design, the analysis relied on medical records. Therefore, it is possible that not all potentially relevant information or factors that may affect patient outcomes were accounted for. Thirdly, limited sample size and relatively small event ratios of individual variables may have affected the accuracy and statistical significance of the regression coefficients. Finally, we did not control for potential confounding factors such as the varying expertise of surgeons and individual patient rehabilitation compliance, which may significantly affect the observed results. All these limitations should be considered when interpreting this study’s results and validated through other randomized controlled trials with longer follow-up periods. We intend to conduct further higher-quality investigations to validate our findings and build a predictive model to help prevent epilepsy recurrence and promote early intervention.

## CONCLUSION

Lesion location and size, timing of epileptic seizures, ICH, hemorrhagic transformation, HHcy, and NIHSS scores were all found to be risk factors for epilepsy recurrence in patients with PSE. The implementation of targeted prevention and control measures in the clinical setting may improve patient prognosis.

### Authors’ contributions:

**XW:** Study design, literature search and manuscript writing.

**HZ, LG, LZ and ZL:** Data collection, data analysis and interpretation. Critical Review

**XW:** Critical Review, manuscript revision and validation.

All authors have read, approved the final manuscript and are responsible for the integrity of the study.
